# Temperature but not leptin prevents semi-starvation induced hyperactivity in rats: implications for anorexia nervosa treatment

**DOI:** 10.1038/s41598-020-62147-z

**Published:** 2020-03-24

**Authors:** Angela Fraga, Marcos C. Carreira, Andrea Gonzalez-Izquierdo, Carlos Diéguez, Miguel López, Emilio Gutiérrez

**Affiliations:** 1 Department Psicología Clínica y Psicobiología, Facultad de Psicología, Universidad de Santiago, Campus Vida, 15782 Santiago de Compostela, Spain; 2 Unidad Venres Clínicos, Facultad de Psicología, Campus Vida, Universidad de Santiago, 15782 Santiago de Compostela, Spain; 3 Lab de Endocrinología Molecular, Instituto de Investigaciones Sanitarias de Santiago de Compostela (IDIS), Complej o Hospitalario de Santiago (CHUS), A Coruña, Spain; 4 CIBER Fisiopatología Obesidad y Nutrición (CIBERobn), Instituto de Salud Carlos III, Madrid, Spain; 5 Department Fisioloxía and Centro de Investigación en Medicina Molecular (CIMUS), Universidade de Santiago de Compostela, Instituto de Investigaciones Sanitarias de Santiago de Compostela (IDIS), Santiago de Compostela, 15782 Spain

**Keywords:** Psychology, Malnutrition

## Abstract

The hypothesis linking hyperactivity with weight loss associated hypoleptinemia in anorexia nervosa gained momentum after a study showing that leptin suppressed semi-starvation induced hyperactivity in rats. Alternatively, ambient temperature is a key modulating factor of activity in semi-starved rats. The aim of the study is to compare the efficacy of leptin with increased ambient temperature in the prevention of hyperactivity in semi-starved rats. 74 Sprague-Dawley male rats were employed in two experiments with the difference residing in the length of baseline. After an extended (28 days), or shorter (14 days) baseline with free access to food and the running wheel, housed at 21 °C, animals were either ad-lib feed or food restricted (60% of food ingested during previous week) and infused with same amount of leptin at 21 °C, 25 °C, or vehicle at 21 °C, 25 °C and 32 °C for a week. Animals housed at 32 °C significantly reduced wheel running and weight loss during food restriction while animals given leptin did not yield no differences in activity or weight loss. Moreover, unlike animals housed at 32 °C, body temperature of leptin infused animals housed at 21 °C was significantly reduced during food restriction. Furthermore, leptin treated rats without a preceding stable pattern of activity displayed a severe dysregulation of circadian rhythm in activity and a collapse of body temperature. Housing temperature plays a more critical role than leptin in the regulation of semi-starvation induced hyperactivity in rats, which may be of relevance for the management of hyperactivity in anorexia nervosa.

## Introduction

Hyperactivity is a characteristic sign in anorexia nervosa (AN) has been described since the first modern portrayals of the illness^1,2^ with a prevalence as high as 80%^3^. Although the relevance of excessive activity was considered a fundamental clinical feature^4^ even before the DSM-III^5^ was published, hyperactivity has traditionally been considered marginal among AN signs^6^, being conceptualized as a mere strategy to burn off calories^7^. However, this conceptualization contradicts research showing that excessive activity often precedes the onset of the disorder^8^, as already pointed and the beginning of XX century^9^.

The negative vicious cycle between diet and exercise is reproduced in two analogous animal models that mimic weight loss and hyperactivity, as well as many others signs in AN; the activity based anorexia (ABA) model^10^, and the semi-starvation induced hyperactivity (SIH) model^11^. Both models focus on the excessive activity of food-restricted rats but whilst ABA underlines the effect of running activity in food intake, that is, self-starvation^12^, SIH focuses on the effects of food restriction on the increase of running activity. The key difference between ABA and SIH lies in the way rats face food restriction. Food restriction in SIH procedure is a restriction in the amount of food served to rats, usually rats receive a daily ration equal to 60% of food consumed during previous ad lib phase, and rats are allowed eat that amount during the 24 hours after food delivery. In contrast, food restriction in ABA consist of a restriction in the time in which rats have access to food. This means that the day that ABA rats are introduced to the food restricted schedule they have a limited time (between 1 h and 1.5 h) to eat as much as they are able. However, given the natural feeding pattern of rats consists of brief visits to the feeder during the hours of darkness with occasional visits during the light period, the food restriction in SIH is more lenient than in ABA^13^. The ABA model is a more demanding model, as a matter of fact 80% of the animals end up being removed from the experiment due to the severe weight loss in order to avoid impending death. On the other hand, the amount of food provided to animals exposed to SIH allows a less substantial weight loss and thus self-starvation is prevented in rats.

Among the different hypotheses put forward to explain the increase in activity in food restricted rats, two focus on the consequences of weight loss; hypoleptinemia and hypothermia. Leptin levels increase with adipose tissue accretion and decreases with loss of adipose mass^14^. Seen from this perspective, hypoleptinemia would trigger foraging behavior due to the disappearance of leptin’s inhibition over the reinforcement effect of physical activity^15–17^. As for, the hypothesis underlining hypothermia, it is suggested that increased activity in calorie restricted animals could be a surrogate thermoregulatory mechanism to maintain body temperature^18^.

Both hypotheses have been studied in the ABA procedure. With respect to leptin, a pivotal study^19^ showed that the peripheral administration of leptin via osmotic minipumps both prevented and reversed hyperactivity. However, despite wheel running reduction was observed, this was not translated into body weight recovery. Moreover, chronic intracerebroventricular (ICV) infusion of leptin successfully prevented excessive running in ABA, although rats had to be withdrawn from the experiment due to hypothermia with a substantial weight loss and a significant lower cumulative food intake than vehicle infused animals^20^. Moreover, acute ICV leptin into the ventral tegmental area (VTA) suppressed wheel running without a negative effect on food intake^21^. Similar results were reported when leptin was bilaterally injected in the VTA. With respect to hypothermia, previous research in rats exposed to ABA has shown that the supply of heat^22–24^ prevented excessive running and weight loss. In this sense, increased ambient temperature (Ta), both reversed excessive activity and improved food intake allowing bodyweight recovery, an effect replicated in male and female animals^25–27^.

To date, the hypothesis linking the increase in activity to hypothermia has not been tested in SIH, and no replication has been published regarding leptin effects on SIH. Thus, the aims of this study are twofold: 1) to replicate the original study performed by Exner *et al*.^19^, and to compare the efficacy of leptin and heightened Ta in SIH model in order to determine if housing temperature and not leptin plays a critical role in the regulation of semi-starvation induced hyperactivity in rats, which may be of relevance for the management of hyperactivity in anorexia nervosa in humans. It is worth noting, the suggestion of keeping patients warm was first recommended by William Gull in his seminal paper where the term anorexia nervosa was coined^28^.

## Materials and Method

### Subjects

Male Sprague-Dawley rats (130–190 g) were acquired from the University Animal Resources Centre. They were kept in the colony room with food and water ad libitum on a 12-hr light – dark cycle (LD, lights on from 0800 to 2000 hours). Ambient temperature set at 21 °C ± 1 °C.

The ethics committee on the use and care of animals of Santiago de Compostela University approved all described procedures (project license 15004/17/002). All experiments were carried out in accordance with Royal Decree 53/2013 of February 1, Law 32/2007 of November 7, and European Communities Council Directive 2010/63/UE of September 22, on the protection of animals used for experimental and other scientific purposes.

### Apparatus

Cages (48 ×31.5 ×47 cm) equipped with a Whatman-type activity wheel (1.12-m circumference 35.7 cm diameter, 10-cm-wide running surface of a 10-mm mesh bounded by clear Plexiglas and stainless steel walls; Panlab Harvard Apparatus, Barcelona) were placed inside wooden incubators (60 × 60 × 60 cm) with polycarbonate roofs, provided with a 150-W heat wave lamp, connected to a thermostat and a probe positioned at the level of the animal, which allowed individual control of Ta. All cages were lined with wood shavings (Poplar wood granulate; Safe Select Fine, Wordwide Headquaters, Germany).

### Procedure

Two studies were performed with the difference residing in the length of the baseline. In both experiments, body temperature and activity transmitters (PTD 4000 E-Mitter; Respironics Mini Mitter Inc.,) were implanted into rats one week prior to the start of the experiment. The rats were allowed seven days to recover and were left undisturbed except for being weighed on alternate days.

On the eighth day, rats were weighed and transferred to running wheel cages and were feed ad libitum (AL) with a standard diet (SAFE A04: 3,1% fat, 59,9% carbohydrates, 16,1% proteins, 3.339 kcal·g^−1^; Augy, France) and given free access to running wheels for 24 hours (Phase I). The duration of this AL phase was of 28 days in the first experiment (see Supplementary Fig. S1A) and 14 days in the second experiment (see Supplementary Fig. S1B and S1C). Ambient temperature in both experiments was set at 21 °C. Animals were weighed between 1030 and 11 hrs before being provided with enough quantity of food. Body weight and food intake were measured daily and running activity and body temperature were recorded every minute.

Phase 2, a food restriction phase (RF) began with rats matched for activity and body weight being assigned to one of three groups. During RF phase rats were given a 60% of the food ingested during the previous week. On Day 0, animals were briefly anaesthetized and osmotic minipumps (model 2001; Alzet Osmotic Pumps Corp) were implanted subcutaneously delivering either 1.29 mg/ml of recombinant rat leptin (supplied by ProSpec-Tany Techno-Gene Ltd.,) or a similar volume of vehicle (phosphate buffered saline) during the RF phase. As indicated in the two digits of the abbreviated group name, during the RF phase Ta remained constant at the same temperature as in the previous AL phase for four groups (21LEP, 21VH, 25 LEP, and 25 VH), whereas Ta was increased from 21 °C to 32 °C for a fifth group (32VH). Furthermore, two additional groups of rats housed at 21 °C were maintained with food ad libitum for a week after completing a 14 days AL phase, and they did not undergo food restriction (Supplementary Fig. S1C).

The animals were sacrificed by decapitation on Day 6 after the weighing routine. Trunk blood was collected into specific tubes (BD Vacutainer) and centrifuged at 3200x g for 15 min at 4 °C to separate the serum. Then serum was stored at -80 °C until assay. Interscapular brown adipose tissue (BAT) was collected, frozen and stored at −80 °C. Unfortunately, a freezer malfunctioning damaged stored blood samples from Experiment 1 and from ad libitum study.

### Surgeries

Both transmitter and osmotic minipumps were implanted under ketamine-xylazine anaesthesia (50 mg/kg, intraperitoneal). The ventral surface of the abdomen (for transmitter) and the dorsal surface of the lumbar area (for osmotic minipump) was shaved and sterilized. A 1 cm skin incision and a cavity were made using blunt dissection. The corresponding device was slipped into the abdominal or the lumbar cavity and finally the incision was sutured with surgical silk.

### BAT Western blotting

A third portion of collected BAT was homogenized in lysis buffer containing protease inhibitor cocktail tablets (Roche Diagnostics), and the protein concentration was determined using the Bradford method (Protein assay dye concentrate, Bio-Rad Laboratories).Ten micrograms of total protein were subjected to SDS-PAGE on 10% polyacrylamide gels, and electro-transferred to polyvinylidene difluoride membranes with a semidry blotter. Membranes were blocked in TBS/Tween with 3% of BSA (Bovine serum albumin, Sigma Aldrich) and probed with antibodies against UCP1 (Abcam) and a-tubulin (Sigma-Aldrich). Membranes were incubated with the corresponding secondary antibody: anti-rabbit for UCP1 and anti-mouse for a-tubulin (DAKO). Detection of proteins was performed with Enhanced chemiluminiscence (ECL) reagents (Pierce ECL Western Blotting Substrate, Cultek) according to the manufacturer’s instructions, exposed to x-ray films, developed and fixed under appropriate dark room conditions. Autoradiographic films were scanned and the band signal were quantified by densitometry using ImageJ-1.33 software (NIH), as formerly shown^29–31^. Values were expressed in relation to a-tubulin. Adobe PhotoShop CC were used to crop and assemble the images for the presentations.

### Serum leptin analyses

Leptin levels were determined using a Rat Leptin ELISA kit (Linco Research St. Charles; Cat #EZRL-83K; sensitivity 0.04 ng/ml).

### Statistical analysis

Data were expressed as mean ± S.E.M. Statistical analysis was performed using SPSS 21.0 software. The statistical analyses performed were repeated measures ANOVA, ONE-Way ANOVA, Student’s t- tests and Chi-square test when appropriate. Significance was established at p < 0.05, two tailed. Bonferroni correction was performed to avoid a type 1 error in all multiple comparisons. When Mauchly’s Test Sphericity was significant, the Greenhouse-Geisser correction was applied.

### Ethical approval

All applicable international, national, and/or institutional guidelines for the care and use of animals were followed.

## Results

### Experiment 1

**Figure 1:**
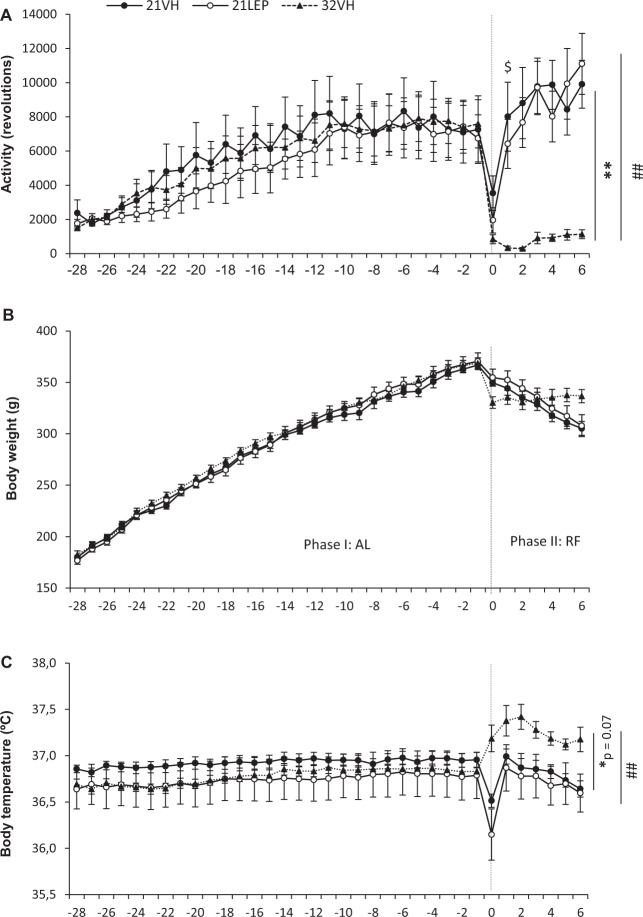
Daily running wheel activity (**A**), body weight (**B**), and body temperature (**C**) from Study 1, during the two last weeks of ad libitum (AL) phase (Day -14 to Day -1), and a food restricted (RF) phase of 7 days of duration. Rats were treated with leptin (open symbols,**○**) or vehicle (closed symbols, ●, ▲) via implanted minipumps during RF phase. Ambient temperature (Ta) during RF was maintained at 21 °C (solid line), as in AL phase, or increased to 32 °C (dotted line). Vertical dashed line separates AL and RF phases. ^*^p < 0.05 and ^**^p < 0.01 for 21VH vs 32VH. ^##^p < 0.01 for 21LEP vs 32VH. ^$^p < 0.05 for 21VH vs 21LEP. Results expressed as mean ± SEM.

Figure 1 shows the evolution of body weight, body temperature, and activity of animals (21LEP, n = 10; 21VH, n = 10; 32VH, n = 11).

There were no changes in running in the last two weeks of the AL phase, F (3.9, 110.9) = 1.662; p = 0.164, as rats showed a stable pattern of activity (Fig. 1A). The implant of minipumps (Day 0) caused an abrupt decrease in activity and body temperature during the remaining light hours of Day 0. For rats housed at 21 °C, running activity recovered to pre-surgery level on Day 1. During the week of RF, a main effect was observed, F (2, 28) = 15,122 p < 0.0001, which showed the running activity of animals housed at 32 °C was less than animals at 21 °C, receiving either leptin or vehicle (both p = 0.001). On the last day of the RF phase (Day 6), the activity of animals housed at 21 °C was eight (21VH) to ten times (21LEP) higher than the activity of warmed rats, and no significant differences were found between groups housed at 21 °C, except on Day 1 when running activity of leptin infused rats was significantly lower than in vehicle treated rats (p = 0.025).

Furthermore, regarding the diurnal rhythm of wheel-running activity, the percentage ratio of Light phase activity/whole day activity [L/(L + D) %, see Table 1] was significantly reduced for 21LEP rats during most of the RF phase in comparison to 21VH and 32VH rats, whilst it was significantly increased for the last day of the RF phase.

**Table 1:** Daily wheel running percentage ratios of light phase /whole day activity - L/(L + D) - across food restriction phase for Study 1. Activity data for first day of food restriction phase has been excluded in the analysis due to the attenuation in activity due to implantation of osmotic minipumps. Abbreviations: ^*a*^Significant differences between 21VH and 32VH. ^*b*^Significant differences between 21LEP and 32VH. ^*c*^Significant differences between 21VH and 21LEP. Chi square test, all differences p < 0.01.

	Study 1						
	FR0	FR1	FR2	FR3	FR4	FR5	FR6
21VH	—	3.2%^*c*^	4.2%^*c*^	8.2%^*c*^	9.8%^*c*^	10.6%^*a*^	9.2%
21LEP	—	1.8%	3.2%	3.6%	5.6%	9.8%^*b*^	14.6%^*b,c*^
32VH	—	14.0%^*a,b*^	14.3%^*a,b*^	6.0%	9.0%	6.3%	8.5%

**Figure 2:**
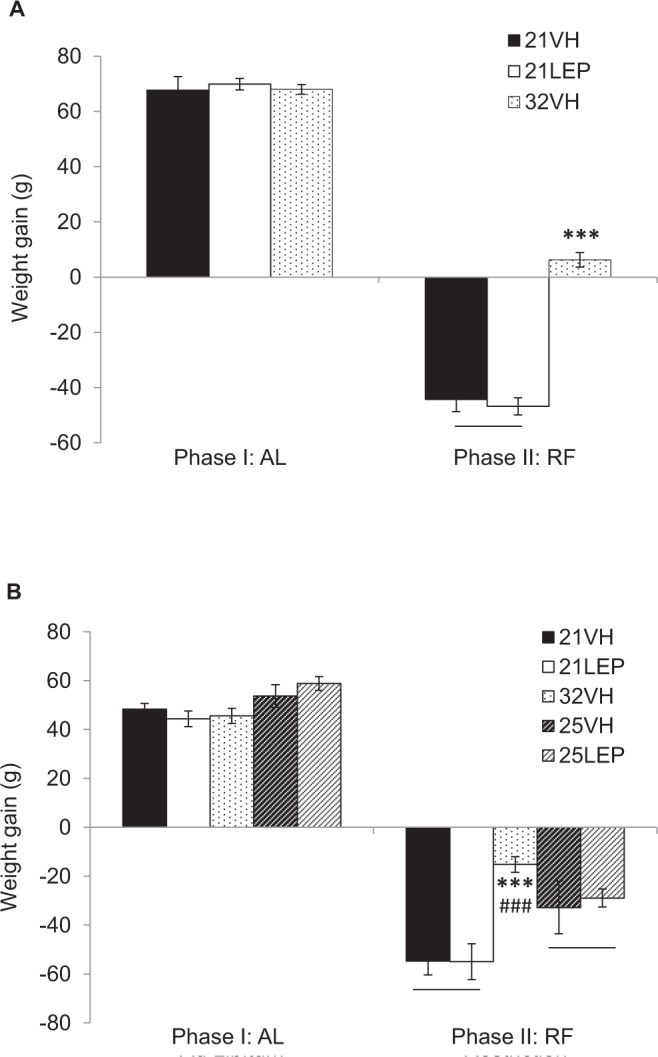
Accumulated weight gain during ad libitum (AL) phase, and restricted feeding (RF) phase for groups receiving leptin or vehicle housed at 21 °C or 32 °C in Study 1 (**A**), and 21 °C, 25 °C or 32 °C in Study 2 (**B**). ^***^p < 0.001 for 21 °C vs 32 °C. ^###^p < 0.001 for 25 °C vs 32 °C. Results expressed as mean ± SEM.

Regarding body weight (Fig. 1B), all animals gained weight over days, F (2.7, 76.5) = 511.837; p < 0.0001, but no significant differences were found between groups in accumulate body weight gain (Fig. 2A) during the last 14 days of the AL phase, (67.70 ± 4.43 g, 69.90 ± 3.11 g, 68.00 ± 2.62 g, for 21VH, 21LEP and 32VH, respectively). During the RF phase body weight evolved differently for the different groups over days, F (2.2, 61.9) = 88.209; p < 0.0001, but whereas groups housed at 21 °C (21VH and 21LEP) lost weight, body weight of 32VH animals significantly increased F (4.4, 61.9) = 33.148; p < 0.001, as depicted on Fig. 2A. No differences in body weight or accumulate body weight gain were found between 21VEH and 21LEP groups.

In regard to body temperature (Fig. 1C) there were no differences between groups for the last two weeks of the AL phase, F (2, 28) = 0.366; p = 0.697. However, during the RF phase there was a main effect for Ta, F (2, 28) = 4.941; p = 0.015, and an interaction effect F (5.2, 73.1) = 2.312; p = 0.05 that was indicative of a decrease in body temperature in 21VH and 21LEP animals (no significant differences between them), while body temperature of 32VH rats increased. Post hoc analysis revealed that differences in mean body temperature were due to differences between 32VH and 21LEP rats (p = 0.002), but differences between 32VH and 21VH rats only approached significance (p = 0.07).

**Figure 3:**
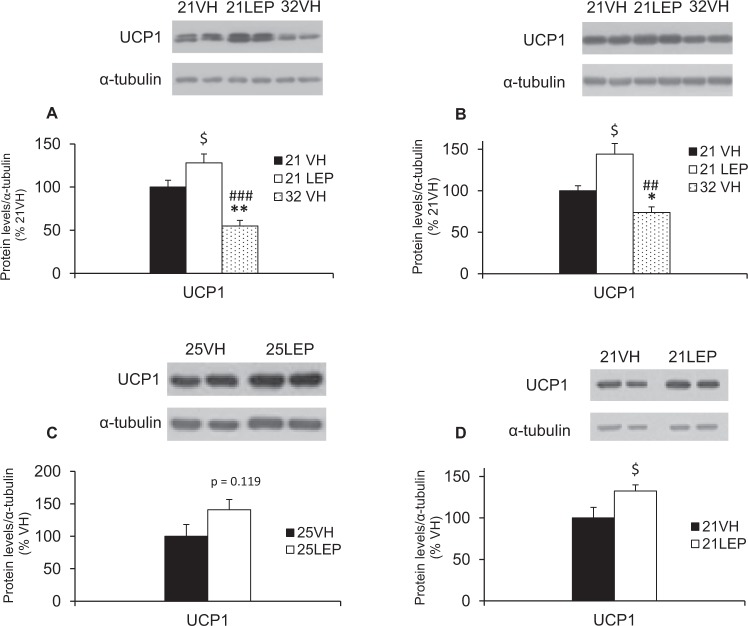
Western blot quantification of UCP-1 protein levels in BAT of rats either exposed to food restriction (RF) or ad libitum-fed (AL) under different ambient temperature (Ta) and treatments (vehicle, VH, or leptin, LEP). (**A**) RF rats housed at 21 °C, receiving leptin or vehicle vs. vehicle-rats housed at 32°C from Study 1. (**B**) RF rats housed at 21° C receiving leptin or vehicle vs. vehicle-rats housed at 32°C from Study 2. (**C**) RF rats housed at 25° C receiving leptin or vehicle. (**D**) AL rats housed at 21°C receiving either leptin or vehicle. ^*^p < 0.05 and ^**^p < 0.01 for 21VH vs 32VH. ^##^p < 0.01 and ^###^p < 0.001 for 21LEP vs 32VH. ^$^p < 0.05 for vehicle vs leptin at 21°C, 25 °C and fed ad libitum. Results are expressed as mean ± SEM. Images of blots have been cropped; uncropped images are shown in Supplementary Figure S6.

As seen in Fig. 3A, protein levels of UCP1 in BAT of 32VH animals were significantly reduced compared to animals housed at 21 °C, receiving either vehicle (21VH, p = 0.001) or leptin (21LEP, p < 0.0001). Otherwise, UCP1 for leptin-treated rats was significantly up-regulated (p = 0.045) when compared to the 21VH group.

### Experiment 2: Short baseline

Given the negative results regarding the poor preventive effect of leptin on wheel running, the efficacy of leptin on reversing wheel running was not studied further. Instead, we shortened baseline by two weeks and added a further two groups housed at 25 °C in order to start the RF phase with a level of wheel running comparable to that shown by animals in Exner *et al*.’s study^16^ since an excessive activity during the AL Phase could be masking the inhibitory effect of leptin on hyperactivity during the FR Phase. On the other hand, another effect of leptin is reduced food intake even in food-restricted animals^32–35^. Therefore, because the presentation and measurement of food consumption in SIH do not allow to detect any appetite-diminishing effect of leptin, food consumption was monitored during the first 90 minutes after being served the daily ration, as typically reported in ABA procedure. This strategy allowed us to verify the proper functioning of leptin.

**Figure 4:**
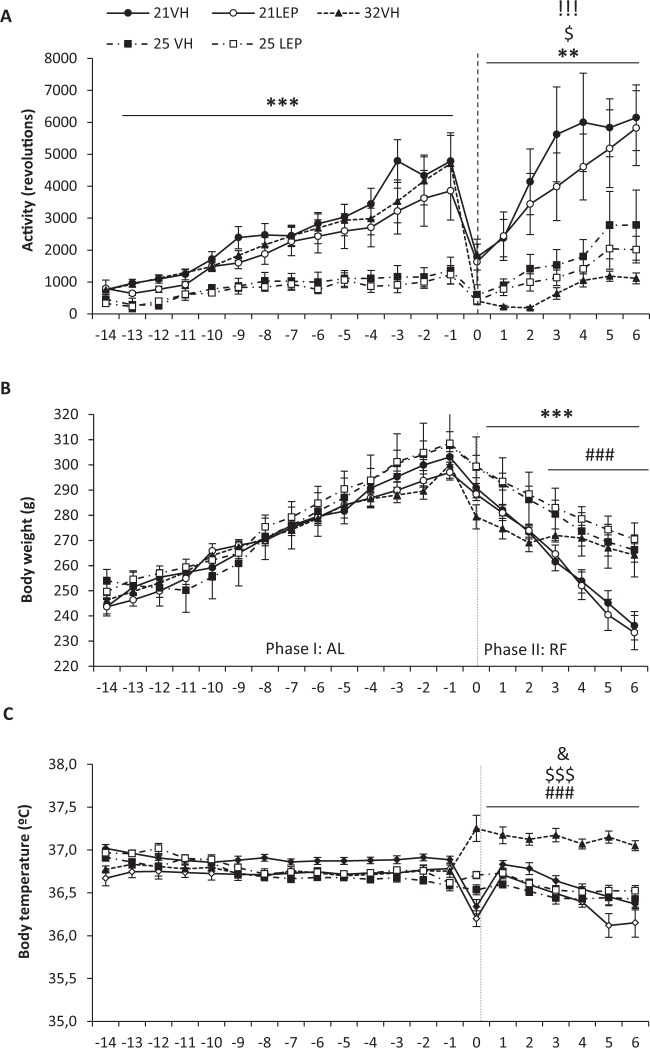
Daily running wheel activity (**A**), body weight (**B**), and body temperature (**C**), during a two weeks ad libitum (AL) phase, and a food restricted (RF) phase of 7 days of duration. Rats were treated with leptin (open symbols, ○, □) or vehicle (closed symbols, ●, ▲, ■) via implanted minipumps beginning on the first day of RF phase. Ambient temperature (Ta) during RF was maintained at 21 °C (solid line) or 25 °C (dot dash line), as in AL phase, or increased to 32 °C (dotted line). Vertical dashed line separates Al y RF phases. ^**^p < 0.01 and ^***^p < 0.001 for 21 °C vs 25 °C. ^###^p < 0.001 for 21 °C vs 32 °C. ^$^p < 0.05 and ^$$$^p < 0.001 for 25 °C vs 32 °C. ^&^p < 0.05 for 21VH vs 21LEP. ^!!!^p < 0.001 for 21VH vs 32VH and 21LEP vs 32VH. Results expressed as mean ± SEM.

With respect to activity (Fig. 4A), there were no significant differences during the AL phase in animals housed at either 21 °C, or 25 °C, but running was threefold less in animals housed at 25 °C than in rats at 21 °C. Also, as in Study 1, minipumps surgery caused decreased running activity and body temperature but pre-surgery levels of activity were soon recovered for animals housed either at 21 °C, and 25 °C, but not for the animals at 32 °C. During the RF phase, animals maintained at Ta21 °C ran more than Ta32 °C rats, which on the last day showed five times less activity than 21VH or 21LEP animals (both p < 0.0001). In comparison, daily running was significantly lower in animals housed at 25 °C Ta than in 21 °C animals (Day 0 to Day 6, p < 0.01), but significantly higher than in Ta 32 °C rats (p < . 05, except for Day 0 and 5).

**Figure 5:**
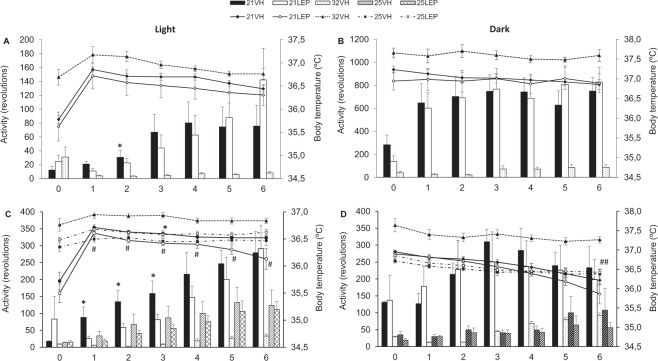
Daily running activity and body temperature for light (left panels) and dark periods (right panels) during food restriction (RF) phase. Top panels (**A,B**) are for rats of Study 1 with a preceding ad libitum baseline of 28 days. Bottom panels (**C,D**) represent data for rats of Study 2 with a preceding ad libitum baseline of 14 days. Rats were treated with leptin (LEP, white symbols ○, □) or vehicle (VH, dark symbols ●, ▲, ■), via implanted minipumps. Body temperature from animals which ambient temperature (Ta) during RF was maintained at 21 °C (solid lines) or 25 °C (dot dash lines), as in AL phase, or increased to 32 °C (dotted line). ^*^p < 0.05 for 21VH vs 21LEP. ^#^p < 0.05 and ^##^p < 0.01 for 21LEP vs 25LEP. Activity and body temperature are presented as mean ± SEM. Legends. Wheel running bars: 21VH, black bars; 21LEP, white bars; 32VH, dotted bars; 25VH, hatched bars; 25LEP, cross-hatched bars.

With respect to the diurnal rhythm of wheel-running activity at variance with Study 1 warming prevented the rise of L/(L + D) % in 32VH rats which was significantly lower than in 21 °C housed rats (Table 2). Furthermore, leptin again significantly reduced the L/(L + D) % of 21LEP during most of the RF phase with respect to 21VH rats, but on last day 21LEP rats significantly exceeded the activity of 21VH rats, reaching 60% of the total daily activity during the light period. Regarding rats housed at 25 °C, the L/(L + D) % of 25LEP rats was significantly reduce during the first half of the RF phase (Table 2), but in the second half leptin again aggravated disruption of the diurnal rhythm of wheel-running activity. Moreover, as depicted Fig. 5C, mean diurnal activity of 21LEP rats was significantly lower in comparison to 21VH rats for Days 1–3 (all p < 0.05).

**Table 2:** Daily wheel running percentage ratios of light phase /whole day activity - L/(L + D) - across food restriction phase for Study 2. Activity data for first day of food restriction phase has been excluded in the analysis due to the attenuation in activity due to implantation of osmotic minipumps. Abbreviations: ^*a*^Significant differences between 21VH and 32VH. ^*b*^Significant differences between 21LEP and 32VH. ^*c*^Significant differences between 21VH and 21LEP. ^*d*^Significant differences between 25VH and 25LEP. Chi square test, all differences p < 0.01.

Study 2							
	FR0	FR1	FR2	FR3	FR4	FR5	FR6
21VH	–	41.0%^*a,c*^	38.7%^*a,c*^	33.9%^*a,c*^	43.1%^*a,c*^	50.8%^*a,c*^	54.5%^*a*^
21LEP	–	12.5%	20.5%	24.6%^*b*^	38.4%^*b*^	47.0%^*b*^	59.5%^*b,c*^
32VH	–	31.2%^*b*^	16.2%	15.4%	21.3%	25.2%	26.3%
25VH	–	53.2%^*d*^	57.6%^*d*^	68.1%^*d*^	67.2%	57.1%	53.6%
25LEP	–	39.0%	49.8%	58.3%	64.1%	62.3%^*d*^	66.6%^*d*^

Concerning bodyweight, there were no differences in accumulate body weight gain (Fig. 2B), or mean body weight (Fig. 4B) during the AL phase. During the RF phase, there was a significant interaction for Days x Ta, (8.5, 72.4) = 10.937; p < 0.0001 with respect to body weight, 32VH animals showed a slower and less pronounced weight loss respect to animals housed at 21 °C and 25 °C, but no differences were observed between leptin and vehicle animals at 21 °C and 25 °C. On the other hand, the decline in body weight of all of animals housed at 21 °C was significantly greater than for Ta 25 °C animals F (1, 25) = 16.928, p < .0001 (Fig. 4B). When the ANOVA was restricted to the last four days of the restriction phase, there were no differences between animals at 25 °C and 32 °C, but there were significant differences among animals housed at 21 °C and 32 °C, F (2, 25) = 9.392; p = 0.001. Likewise, there were significant differences in accumulated weight gain, with weight loss in the 32VH group being lower than in animals housed at 21 °C and 25 °C (p < .001 in both cases, see Fig. 2B), but no differences were found between vehicle and leptin groups at 21 °C, or 25 °C.

Regarding body temperature (Fig. 4C), there were no differences among groups during the AL phase. During the RF phase, 32VH animals had significantly higher body temperature than those housed at either 21 °C or 25 °C (p < 0.0001 for both groups). Among animals housed at 21 °C and 25 °C, only 21LEP animals showed a lower body temperature than 21VH (p = 0.03). However, as shown in Fig. 5C, differences among leptin treated animals were more clearly limited to light hours as body temperature of 25LEP was significantly higher than that of 21LEP animals for Days1 to 6 (p = 0.05). During the dark phase (Fig. 5D) only on Day 6 was body temperature significantly lower in 21LEP than 25LEP animals.

Regarding food intake, (Supplementary Fig. S2A) food consumption for the first 90 minutes during the RF phase revealed that rats housed at 32 °C ate significantly less than rats housed at 21 °C (both p < 0.0001). Moreover, food intake for 21LEP was significantly lower (p = 0.01) than for 21VH animals during the first 5 days of the RF phase (Day 0 to 4).

With respect to serum leptin levels (Supplementary Fig. S2B), there were differences among groups in circulating leptin levels F (2, 23) = 7.228, p = 0.004. Post hoc analyses revealed that the leptin level was higher for the 21LEP group in comparison to 21VH, t (12) = 3.011, p = 0.011, and 32VH, t (18) = 2.341, p = 0.031, demonstrating the efficacy of the treatment. Moreover, 32VH rats showed higher serum leptin levels than 21VH rats, t (16) = 2.831, p = 0.012.

As for the protein levels of UCP1 in BAT of 32VH animals, these were significantly lower as compared to animals housed at 21 °C, receiving either vehicle (21VH, p = 0.031) or leptin (21LEP, p = 0.001), whilst 21LEP levels were up-regulated (p = 0.026) with respect to 21VH group (Fig. 3B). The comparison between animals housed at 25 °C and 25VH showed differences in protein levels of LEP25 only approached significance (p = 0.119, as shown in Fig. 3C).

### Active ad libitum rats

In order to determine the effect of leptin on food intake, body weight, running activity, and body temperature, a group of rats (n = 15) housed at 21 °C were also studied for a 21-day period. After fourteen days of AL baseline, as in Study 2, two groups of rats matched for pretreatment activity and body weight and were continuously infused with either leptin 1.29 mg/ml (n = 8) or vehicle (n = 7) for seven days.

There were no differences between groups in wheel running during the AL phase. As in Exp. 1 and 2, there was a decrease in activity and body temperature caused by the implanting of minipumps. Leptin seemed to facilitate wheel running (Supplementary Fig. S3A), but no differences were found in running activity during the week rats were infused either with vehicle or leptin, F (1, 13) = 0.513; p = 0.486.

In terms body weight, no differences were found in body weight or accumulated body weight gain during the AL phase, and after implanting minipumps there were no differences either in body weight, F (1, 13) = 0.446; p = 0.516, or in accumulated weight gain, t (13) = 1.036; p = 0.319 (Supplementary Fig. S3B and Supplementary Fig. S4B).

With respect to body temperature, (Supplementary Fig. S3C), there were no differences between groups either during the first two weeks, or in the third week between leptin and vehicle treated rats. As for food intake (Supplementary Fig.u S4A) there were no differences between groups during the AL phase. However, during the third week leptin treated rats ate less than vehicle treated rats, F (1, 13) = 5.560; p = 0.035. Notwithstanding, reduced food intake of leptin treated rats was not reflected in differences in body weight gain during the third week in comparison to vehicle treated rats (Supplementary Fig. S3B).

Finally, regarding protein levels of UCP1 in BAT, these were significantly higher, t (13) = −2.285; p = 0.040 in leptin infused rats, as shown in Fig. 3D.

## Discussion

Our findings provide evidence that increased Ta is more effective than leptin in preventing the increase in running activity and weight loss. Moreover, this report demonstrates the protective role of increased Ta in preventing hypothermia in food-restricted rats, which strengthens the evidence that temperature is a key factor in semi-starved rats.

It is intriguing that leptin treatment did not prevent wheel running as previously reported^19^, and only had a brief and transient effect on reducing wheel running during the light period of the light/dark cycle. However, leptin had no significant effect on total daily wheel-running activity, and at the end of the RF phase the increase in activity of leptin infused rats during light hours surpassed the activity of vehicle rats. Nevertheless, in line with results reported elsewhere^19^ leptin facilitated wheel running in ad libitum-fed running rats, but again at variance with this study leptin did not produce significant differences in body weight in ad libitum-fed running rats. The use of recombinant rat leptin, as in our study, instead of recombinant mouse leptin is more appropriate, although molecules are 95.9% similar and no significant differences were detected in relation to food intake, body weight or body temperature^36^. Therefore, this discrepancy in the result may be partially due to the different timing in food delivery; three hours after the beginning of the light period in our study vs. one hour before the beginning of the dark period in other studies^19^. As a nocturnal animal, rats are more active and eat during the night so mistimed eating has metabolic consequences^37,38^.

Moreover, there is a substantial difference in wheel running between our study and the former report^19^, where a maximum activity of 4800 wheel turns the last day of the RF phase was reported (a level of baseline activity three times higher); in contrast our animals ran over 11.000 turns, only 1.4 times baseline activity. There is no simple answer to these differences in activity and they cannot be attributed to the different strain of rats used in our study, as there are not reported differences in circadian wheel running between Sprague Dawley and Wistar rats^39^. Furthermore, in all three studies reviewed, the reported level of activity during ad libitum feeding was similar for the three different batches of rats employed and is in line with the paper that served as the basis for the SIH procedure^40^. A likely explanation for differences in wheel running during the ad libitum baseline resides in the two Celsius degrees of difference in Ta between the two studies, as Ta is a potent modulator of activity in laboratory rats^18^. In fact, the low baseline activity shown in the Exner *et al*. study^19^ parallels ad libitum-fed baseline activity of rats housed at 25 °C, which is coherent with activity reported by previous research performed at this Ta^11^. However, even in these Ta conditions, contrary to a previous report^19^, leptin worsened dysregulation of the diurnal rhythm of wheel running. Future studies may explore the possibility that leptin effects would require moderate achiever rats that do not attain high stable levels of spontaneous activity^41^.

Adaptative thermogenesis is essential to maintain body temperature under sub-thermoneutral ambient temperature and is one of the physiological responses that expends most energy^42^. Food restriction influences BAT thermogenesis throughout the reduction in UCP1 levels^43^ while physical activity can increase, reduce or not alter it in rodents^44^. In addition, leptin increased adaptive heat production too^45^. Thus, although UCP1 expression in BAT was significantly down-regulated in 32VH rats, leptin increased UCP1 in BAT with respect to vehicle-rats but was not associated to increased body temperature nor higher weight loss either under ad libitum or restricted feeding housed at 21 °C^46^. Considering that 21 °C represents a mild cold stress even for ad libitum-fed animals^47^, the reduced energy intake during the RF phase is clearly insufficient to compensate for the energetic cost associated with the defense of body temperature summed to the cost of wheel running. Although, animals in Study 1 housed at 21 °C almost doubled wheel running in comparison to animals in Study 2, they showed a better preservation of body weight, body temperature and lower L/L + D ratio during the RF phase, irrespective of treatment. However, the four weeks’ ad libitum-fed baseline in Study 1 allowed for a better adaptation of the rats to the wheel. Furthermore, as food received during the RF was 60% of food ingested during previous week, higher food consumption at the end of AL phase in Study 1 provided these animals with an extra 5 grams (17.55 kcal/g) of food daily during the RF phase (Supplementary Figure S5). However, these differences among animals at 21 °C between Study 1 and 2 were attenuated in animals housed at 25 °C, and disappeared in animals housed at 32 °C. Thus, better body weight preservation in warmed animals was linked to reduced energy expenditure, as we have described in a previous pair-feeding study where thermoneutrality (32 °C) was more influential for body weight maintenance than food availability in food restricted rats^27^.

## Conclusion

Considering the subtle and transitory effect of leptin on activity together with its adverse effects on food intake and body temperature^20^, one must be cautious in drawing conclusion regarding the potential utility of leptin in the treatment of AN patients^48,49^. Especially when the association between hypoleptinemia and hyperactivity in AN is not so linear as it was previously thought but also depends on age, duration and stage of the disorder^50^. Thus, at the beginning of the disorder, leptin levels could trigger foraging behavior increasing physical activity, which is maintained until an adaptation to these low levels of leptin occurs and activity levels would not be influenced anymore. Moreover, studies with metreleptine (a synthetic analogue of leptin) in thin women who perform vigorous exercise and with hypoleptinemia, showed a reduction in adipose tissue even when the dose was carefully controlled to avoid weight loss^51,52^. On the other hand, increased Ta effectively prevents hyperactivity, weight loss and hypothermia in rats exposed to SIH procedure.

Bearing in mind the current absence of effective treatments for AN^53–55^, the findings of this study reinforce previous research performed with the ABA procedure^25–27^, and call for translational research exploring the therapeutic utility of heat in the treatment of hyperactivity in AN^56^. Notably, providing patients with a warming environment of 32 °C has also shown to be useful in reducing postprandial anxiety in AN patients^57^, strengthening previous evidence of the beneficial effect of adding active warming in the treatment of hyperactivity in AN patients^58–62^.

## Supplementary information

Supplementary Figures.
