# Supplementary material for: Temperature but not leptin prevents semi-starvation induced hyperactivity in rats: implications for anorexia nervosa treatment
Source: Sci Rep. 2020 Mar 24;10:5300. doi: 10.1038/s41598-020-62147-z (PMC7093431; doi:10.1038/s41598-020-62147-z)

# Temperature but not leptin prevents semi-starvation induced hyperactivity in rats: implications for anorexia nervosa treatment

Angela Fraga[1], Marcos C Carreira[3,4], Andrea Gonzalez-Izquierdo[3,4], Carlos Diéguez[4,5], Miguel López[4,5], Emilio Gutiérrez[1,2]

[1] Dept. Psicología Clínica y Psicobiología, Facultad de Psicología, Universidad de Santiago, Campus Vida, 15782 Santiago de Compostela, Spain. [2] Unidad Venres Clínicos, Facultad de Psicología, Campus Vida, Universidad de Santiago, 15782 Santiago de Compostela, Spain. [3] Lab de Endocrinología Molecular, Instituto de Investigaciones Sanitarias de Santiago de Compostela (IDIS), Complejo Hospitalario de Santiago (CHUS), A Coruña, Spain. [4] CIBER Fisiopatología Obesidad y Nutrición (CIBERobn), Instituto de Salud Carlos III, Madrid, Spain. [5] Dept. Fisioloxía and Centro de Investigación en Medicina Molecular (CIMUS), Universidade de Santiago de Compostela, Instituto de Investigaciones Sanitarias de Santiago de Compostela (IDIS), Santiago de Compostela, 15782, Spain.

Corresponding author: Emilio Gutierrez, Dept. Psicología Clínica y Psicobiología, Facultad de Psicología, Universidad de Santiago, Campus Vida, 15782, Santiago de Compostela, Spain. Phone: +34 881 813730. e-mail: emilio.gutierrez@usc.es.

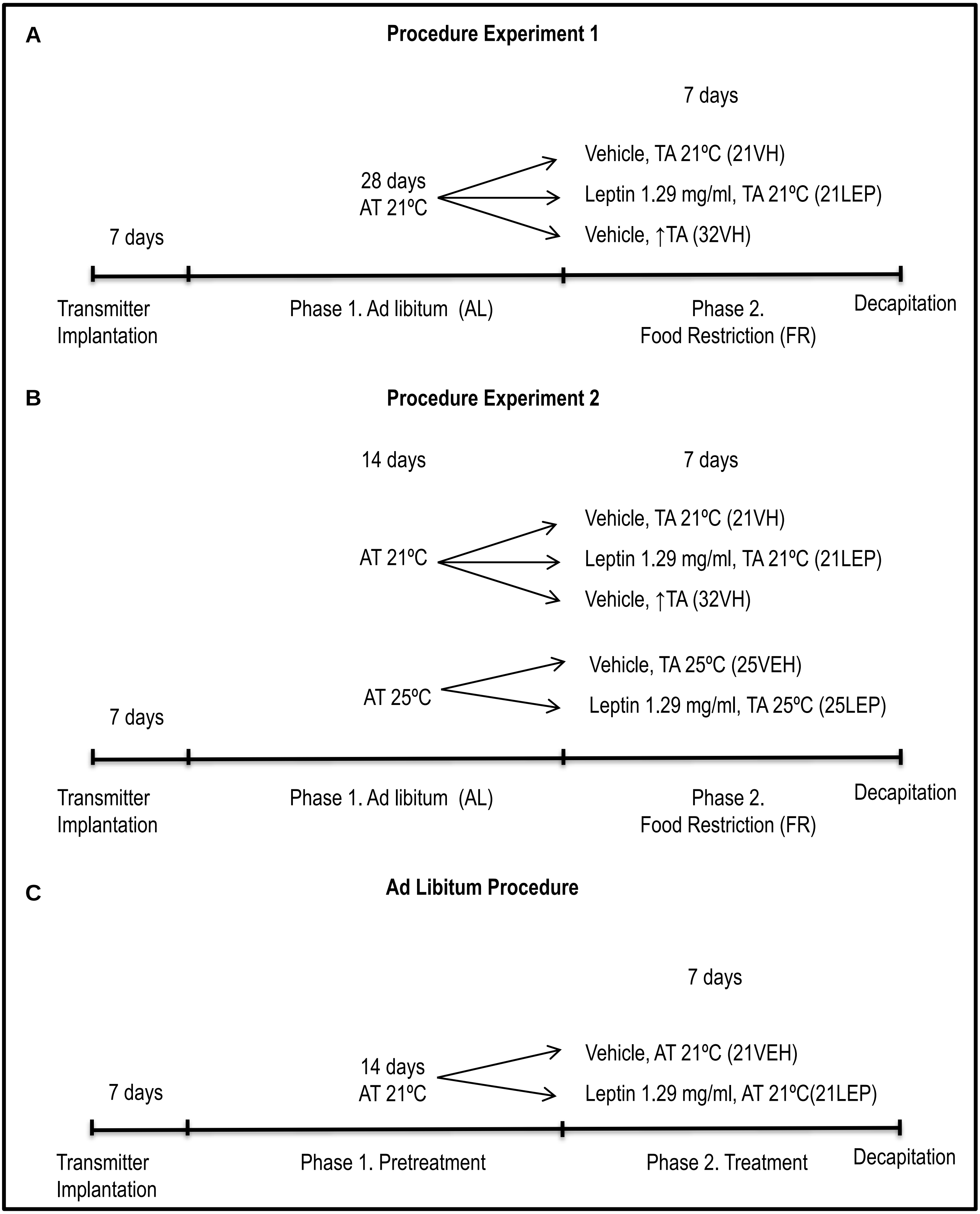


**Supplementary Figure S1.** Diagram of the procedure for Experiment 1 (A), for Experiment 2 (B) and for Ad libitum fed animals (C).

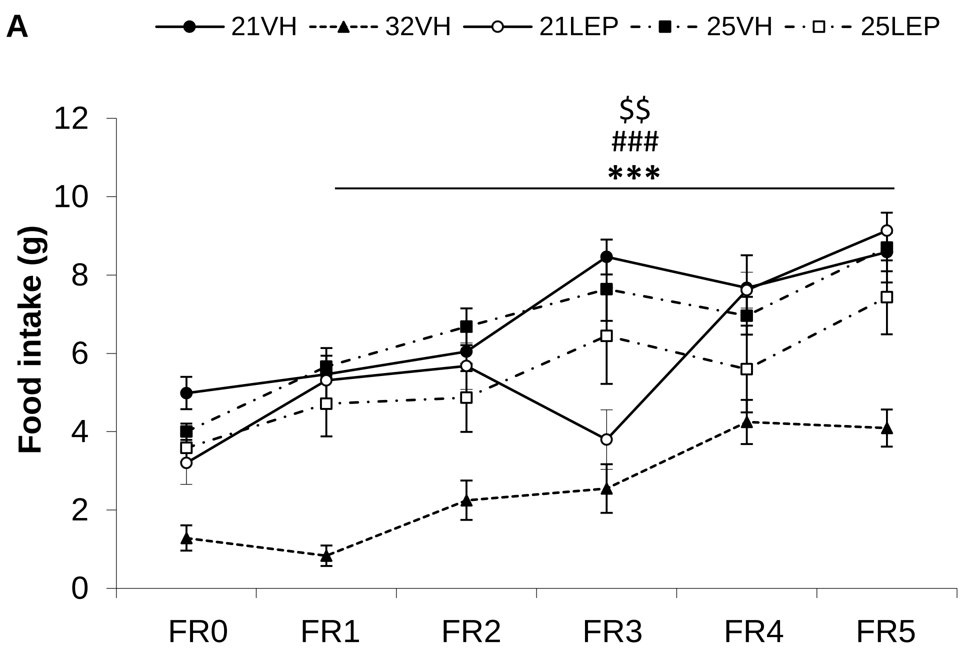


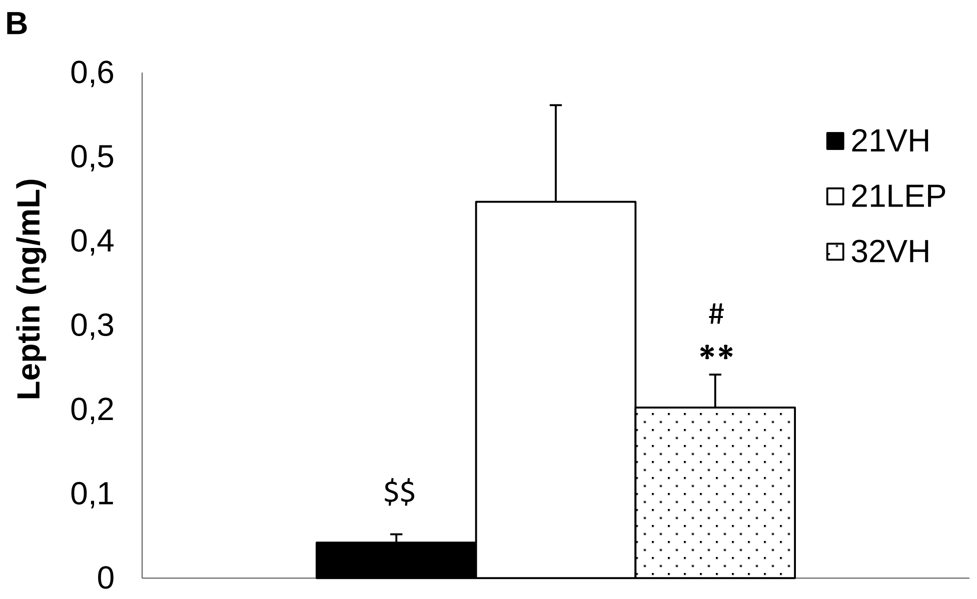


**Supplementary Figure S2**. A) Daily food intake during 90 minutes after food rations served to animals in Study 2 throughout seven days of food restriction (RF) phase. Rats were treated either with leptin (open symbols, ○, □) or vehicle (closed symbols, ●,▲,■) via implanted minipumps beginning on Day O. Ambient temperature (AT) during RF was maintained at 21ºC (solid line) or 25ºC (dot dash line), as in AL phase, or increased to 32ºC (dotted line). B) Plasma leptin levels, ng/ml (mean ± SEM) of animals in the Study 2 housed at 21ºC, receiving leptin or vehicle vs. vehicle-rats housed at 32ºC. $^{**}$ $p < 0.01$ and $^{***}$ $p < 0.001$ for 21VH vs 32VH. $^{\#}$ $p < 0.05$ and $^{\#\#\#}$ $p < 0.001$ for 21LEP vs 32VH. $^{\$\$}$ $p < 0.01$ for 21VH vs 21LEP. Results expressed as mean ± SEM.

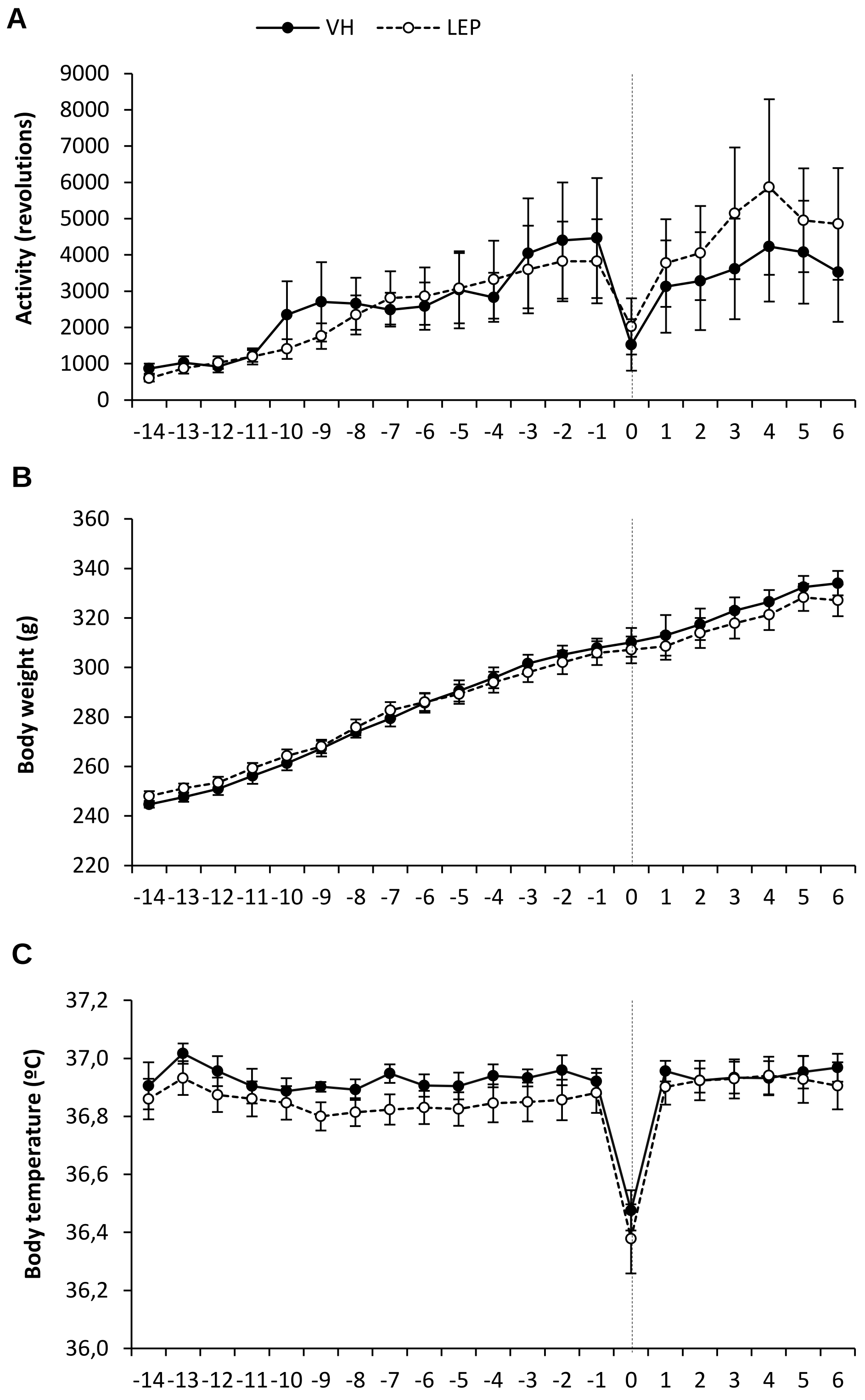


**Supplementary Figure S3**. Daily running wheel activity (A), body weight (B), and body temperature (C), during a three-weeks period of ad libitum feeding. During third week rats were treated with leptin (open symbol,○) or vehicle (closed symbol, ●) via implanted minipumps. Ambient temperature (AT) during third week was maintained at 21ºC) as in the preceding two weeks. Vertical dashed line indicates start of leptin or vehicle infusion. Results expressed as mean ± SEM.

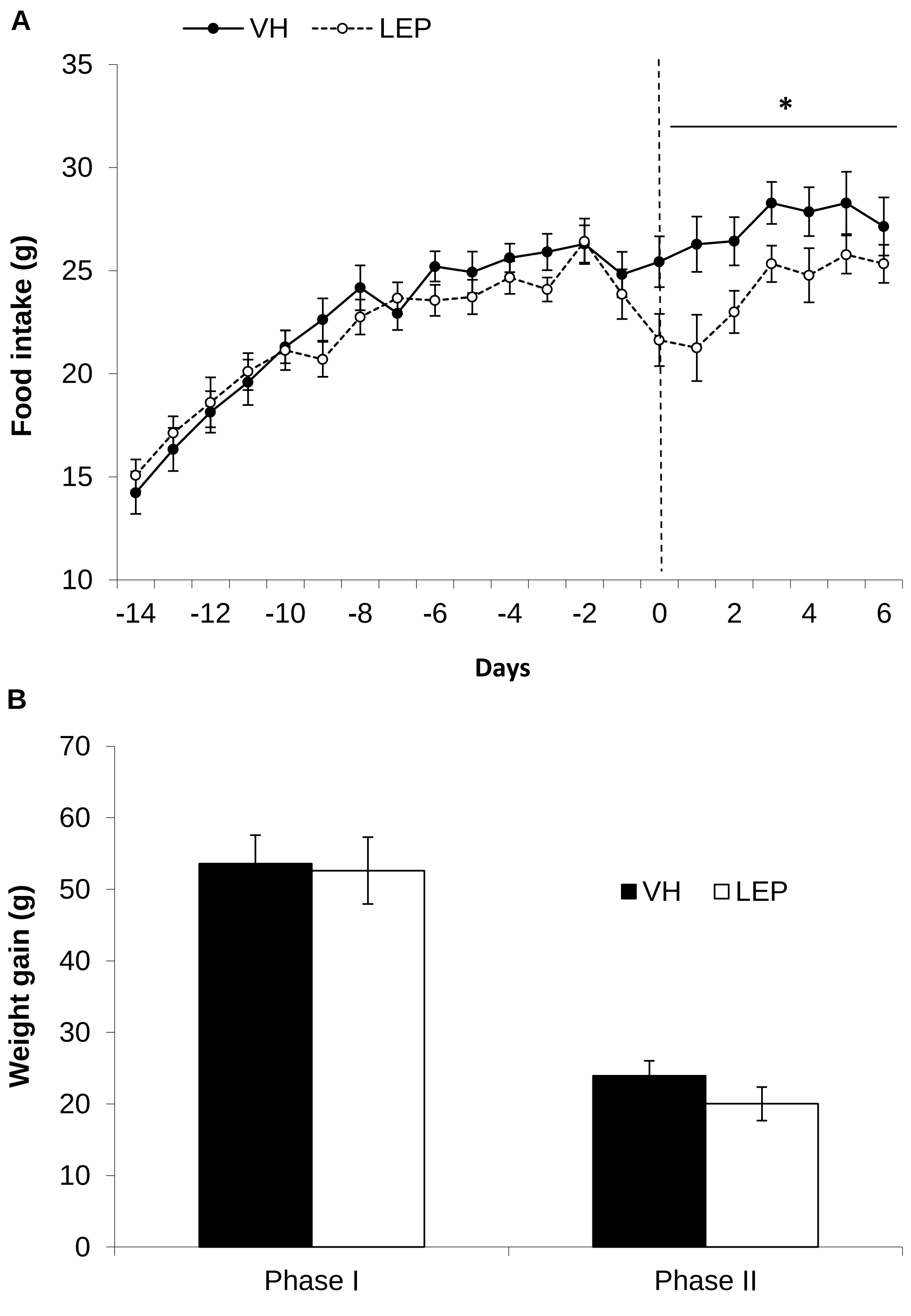


**Supplementary Figure S4**. Daily food intake (A) and accumulated weight gain (B) in ad libitum fed animals during a three weeks period. During third week rats were treated with leptin (open symbol,○) or vehicle (closed symbol, ●) via implanted minipumps. Ambient temperature (AT) during third week was maintained at 21ºC as in the preceeding two weeks. Vertical dashed line indicates start of leptin or vehicle infusion. * $p < 0.05$ for vehicle vs leptin. Results expressed as mean ± SEM.

| | | Phase AL | Phase FR |
|---|---|---|---|
| **Experiment 1** | | 108.38 ± 1.89 | 65.03 ± 1.13 |
| **Experiment 2** | AT 21ºC | 79.13 ± 1.27 | 47.48 ± 0.76 |
| | AT 25ºC | 73.97 ± 2.49 | 44.38 ± 1.49 |

**Supplementary Figure S5.** Overall energy intake during the last week of the Phase AL and daily energy intake during the Phase FR as kcal/g for both studies. Results expressed as mean ± SEM

**Supplementary Figure S6**. Uncropped Western Blot images

**Figure 4A**

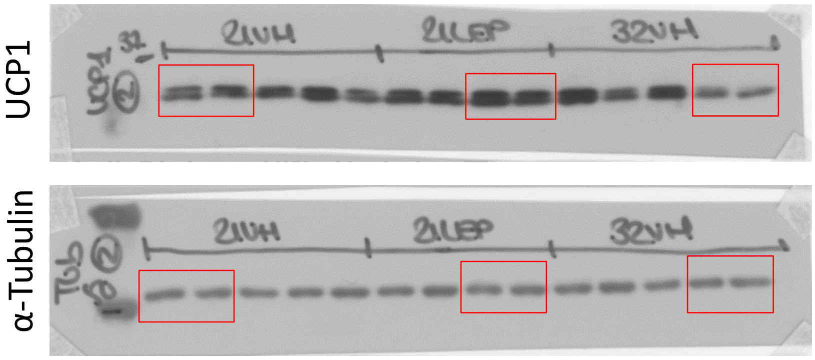


**Figure 4B**

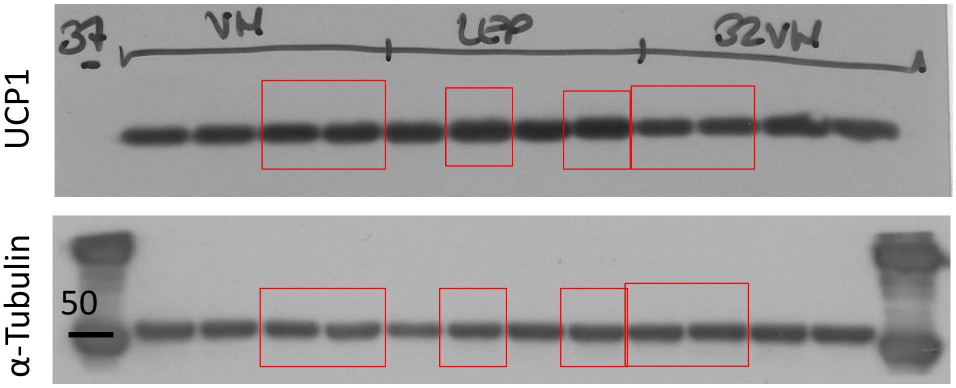


**Figure 4C**

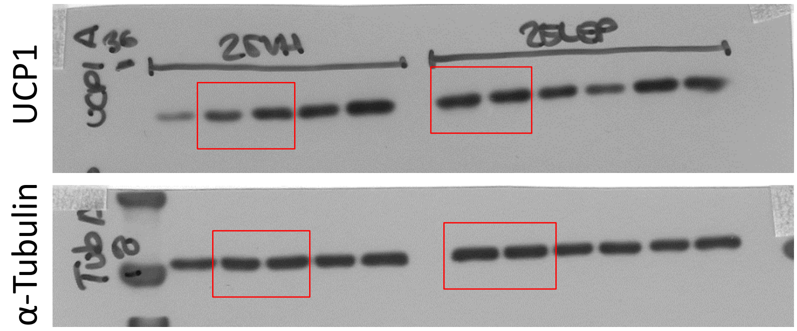


**Figure 4D**

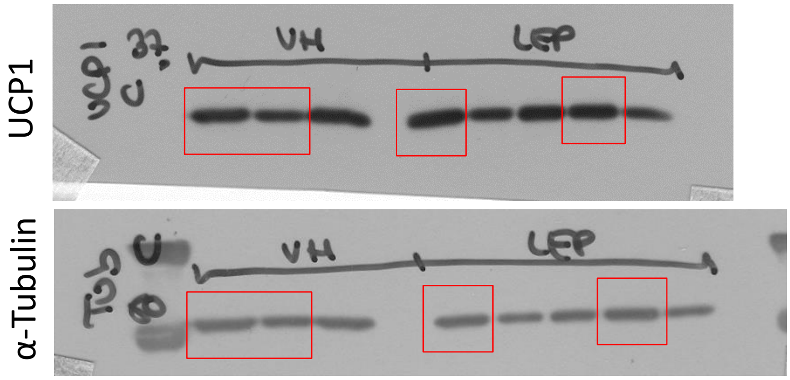

Supplement: Supplementary file 1 — Supplementary Figures. [file 41598_2020_62147_MOESM1_ESM.pdf]
